# Prenatal Diagnosis of Holt–Oram Syndrome With a Novel Mutation of *TBX5* Gene: A Case Report

**DOI:** 10.3389/fped.2021.737633

**Published:** 2021-10-19

**Authors:** Guan-nan He, Xue-yan Wang, Min Kang, Xi-min Chen, Na Xi, Jing Zhao, Xi Chen

**Affiliations:** ^1^Department of Ultrasound, Women and Children's Hospital of Sichuan, Chengdu, China; ^2^Department of Prenatal Diagnosis, Women and Children's Hospital of Sichuan, Chengdu, China; ^3^Department of Radiology, Women and Children's Hospital of Sichuan, Chengdu, China

**Keywords:** Holt-Oram syndrome, *TBX5* gene, novel mutation, prenatal diagnosis, case report

## Abstract

**Background:** Holt–Oram syndrome (HOS) is an autosomal dominant disorder caused by mutations of *TBX5* gene.

**Case presentation:** We report a fetus with HOS diagnosed sonographically at 23 weeks of gestation. The fetal parents are non-consanguineous. The fetus exhibited short radius and ulna, inability to supinate the hands, absence of the right thumb, and heart ventricular septal defect (VSD), while the fetal father exhibited VSD and short radius and ulna only. Fetal brother had cubitus valgus and thumb adduction, except for VSD, short radius and ulna. The pregnancy was terminated. Whole-exome sequencing (WES) revealed a novel mutation in the *TBX5* (c.510+1G>A) in the fetus inherited from the father. The variant (c.510+1G>A) occurs at splice donor and may alter *TBX5* gene function by impact on splicing. It was not previously reported in China.

**Conclusion:** Our case reported a novel mutation in *TBX5*, which expanded the known genetic variants associated with HOS.

## Introduction

Holt–Oram syndrome (HOS) is an autosomal dominant disorder, approximately affecting 1/100,000 live births, which is characterized by upper-limb defects, congenital heart malformation, and cardiac conduction disease (1, 2). The mutations in *TBX5* gene that cause HOS are located on chromosome 12q24 (3). *TBX5* is a member of the T-box family, which plays a vital role in cell-type specification, morphogenesis, and organogenesis (4). Molecular studies have certified that the substantial abnormalities in both the limbs and heart are predicted to be caused by null alleles (5). Mutations within *TBX5* gene include frameshift, splice-site mutations, or nonsense, leading to dysfunctional proteins and resulting in haploinsufficiency, as described (6–8). Vanlerberghe et al. demonstrated that among the identified 49 novel *TBX5* variants, 87% were point changes, including 10% splice site, 14% missense, 26% frameshift, and 37% nonsense variants. Besides, two novel variants responsible for an extended *TBX5* protein were found: one familial case with c.1303delC (Leu435Trpfs^*^147) mutation and one sporadic fetus with c.1346delA (Gln449Argfs^*^70) mutation (9). Although splicing site mutations in *TBX5* gene have been previously reported, there no cases of HOS diagnosed by prenatal ultrasound in China. Here, we report a fetus diagnosed with HOS sonographically at 23 weeks of gestation. The fetus was caused by a heterozygous *TBX5* variant in the splice donor, which was inherited from the paternal variant (c.510+1G>A).

## Clinical Report

**Figure 1:**
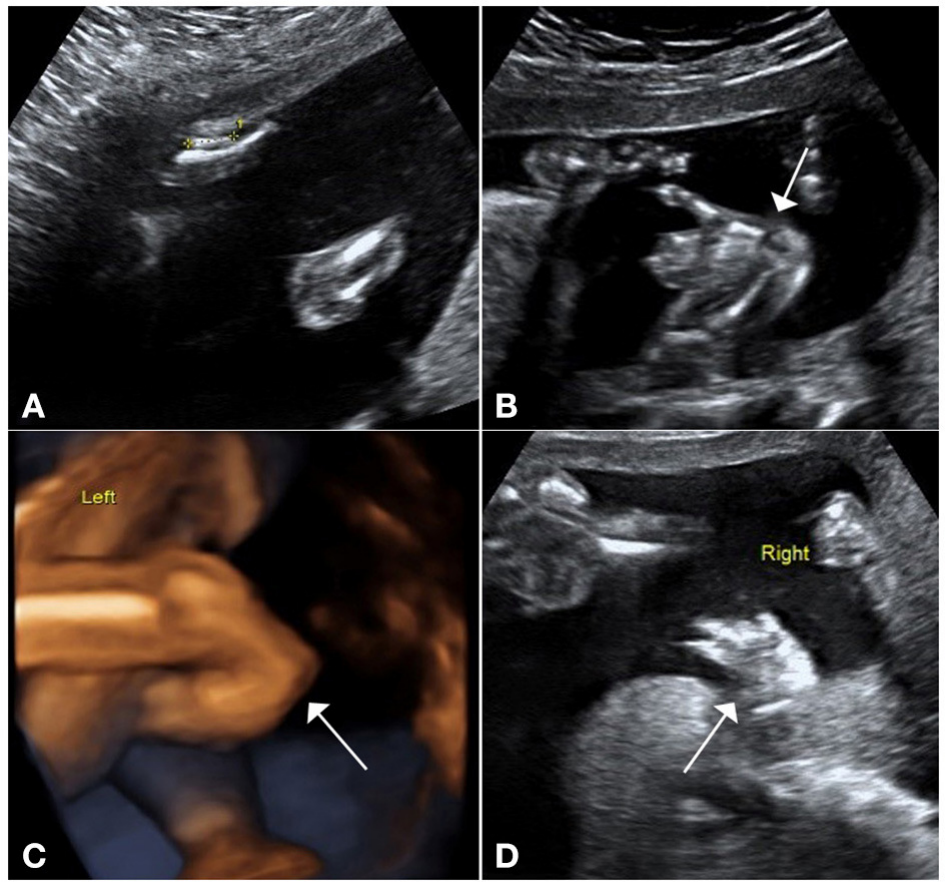
Ultrasound pictures demonstrate upper-limb malformations. **(A)** Ultrasound demonstrates shortness of the radius and ulna, especially the radius. **(B)** Ultrasound and **(C)** 3D views show that the hands had the inability to supinate. **(D)** Right thumb was missing in ultrasound imaging.

**Figure 2:**
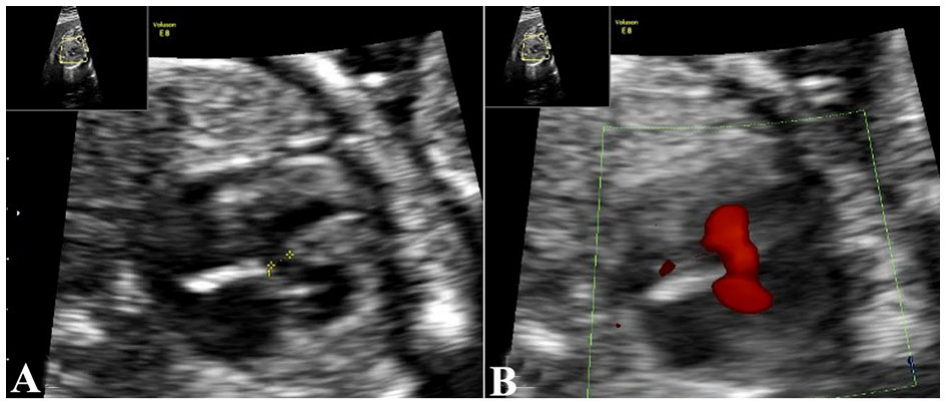
Echocardiography demonstrates cardiac malformations. **(A)** Echocardiography demonstrates a 0.21-cm interruption of continuity in the ventricular septal membrane. **(B)** trans-septal blood flow signals at the VSD. VSD, ventricular septal defect.

A 34-year-old pregnant woman who was at 23 weeks' gestation was referred to our prenatal diagnosis clinic, as fetal skeletal and cardiac abnormalities were diagnosed by another hospital. Her family history and past health were unremarkable. Fetal ultrasonography demonstrated shortness of the radius and ulna, especially the radius (Figure 1A). Both hands had the inability to supinate (Figures 1B,C), and the right thumb was missing in ultrasound imaging (Figure 1D). Fetal echocardiography detected fetal cardiac tissues with ventricular septal defect (VSD), which exhibited an ~0.21-cm interruption of continuity in the ventricular septal membrane, and the signals of trans-septal blood flow were detected at the VSD (Figures 2A,B). Based on this ultrasonic diagnosis, a tentative diagnosis of HOS was made. Next, whole-exome sequencing (WES) was performed for further confirmation. Fetal amniotic fluid cells were collected by amniocentesis for WES; and WES was also performed on family members. Further verification of false-positive text by Sanger sequencing was performed to exclude false-positive variants.

**Figure 3:**
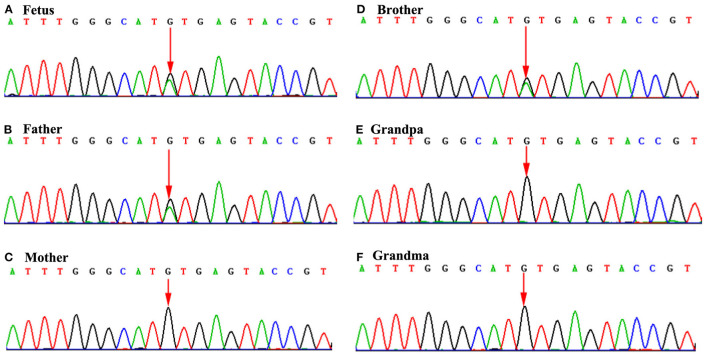
Sequence chromatograms of *TBX5* gene in the family members. **(A)**, **(B)**, and **(D)**: Mutation analysis revealed c.510+1G>A alteration in splice donor of TBX5 (**A**: fetus; **B**: father; and **D**: brother). **(C)**, **(E)**, and **(F)**: Mother, grandpa and grandma carries the normal TBX5 gene. ^*^The arrow of **(A)**, **(B)**, and **(D)** indicates the mutation site of TBX5 (c.510+1G>A) alteration in splice donor. But the arrow of **(C)**, **(D)**, and **(F)** indicates no mutation.

The fetus was the second child of non-consanguineous Chinese parents. The fetal brother (same parents) had cubitus valgus, thumb adduction, short radius and ulna, and VSD, while the father had short radius and ulna and VSD only. But the mother did not have any signs of limb or heart abnormalities. The WES revealed a heterozygous mutation in the fetus (Figure 3A). The variant, c.510+1G>A (RefSep NM_000192: c.510+1G>A), was inherited from the father, and mother is normal (Figures 3B,C). The variant in *TBX5* gene was also found in the brother (Figure 3D), but not in grandfather and grandmother (Figures 3E,F). The variant occurs at splice donor. Further analysis by Human Splicing Finder system version 3.1 (HSF3.1) demonstrated that the mutation most probably influenced splicing, with the variation in the mutated sequence estimated to be 61.14, and variation for the wild-type sequence being 87.97 (ΔCV: −30.5%). According to clinical phenotype and genetic analysis, the case was diagnosed with HOS. However, follow-up data on the fetus were not available, because the pregnancy was terminated by labor induction.

## Discussion

In this case report, we describe a fetus with heterozygous variants in *TBX5* gene (c.510+1G>A). The fetus clinically exhibited typical *TBX5* phenotypes, manifesting as short radius and ulna, inability to supinate the hands, absence of the right thumb, and heart VSD. Based on these clinical manifestations and genetic analysis, the fetus was prenatally diagnosed with HOS.

HOS can be vividly characterized by heart disorders and deficiencies in hand structure (10, 11). There is a variable expression of both the cardiac defects and skeletal. Secundum-type atrial septal defect (ASD) and VSD are the most common heart defects, and the most commonly affected structure in skeletal abnormalities is thumb, which can be triphalangeal, hypoplastic, or completely absent (5). In our report, the fetus presented with several common features, such as shortened radii, absence of the right thumb, and VSD. The abnormal upper limbs were also usually associated with restriction of supination and pronation in the forearm. Our report also revealed the fetus's inability to supinate the hands, which was in accordance with previous reports (12, 13). Additionally, the severity of the deformity may also vary between affected members of the same family. Offspring may inherit both cardiac defects and skeletal from parents with skeleton deformity only (14). In our case, the father had shortness of the radius and ulna and VSD only. But the fetus exhibited inability to supinate the hands and absence of the right thumb, and the fetal brother had cubitus valgus, thumb adduction, shortness of the radius and ulna, and VSD. A similar study reported on a fetus with HOS diagnosed sonographically at 13 weeks of gestation. The fetus exhibited serious bilateral upper-limb malformations, type B interrupted aortic arch, and VSD, while the mother had bilateral upper-limb malformations only. However, it lacks genomic analysis (14). Atik et al. reported that an 8-year-old male diagnosed as HOS presented with upper-extremity abnormalities and a chest deformity and has a history of VSD, and his mother also exhibited deformation in both hands and forearms. But molecular analysis of the fetus (same parents with the 8-year-old male) was normal for *TBX5* gene in the 13th week of pregnancy (3). This also reflects the characters of high penetrative and variable intrafamilial and interfamilial clinical expression of this autosomal dominant disorder.

To date, variants in *TBX5* gene (chromosome 12q24) are the only known cause of HOS (15). *TBX5* is a transcription factor essential for upper-limb formation and cardiac development; there are nine genes in this gene, which include two to nine coding proteins (4). Mutations in *TBX5* genes are associated with skeletal and cardiac developmental disorders. According to a research report, more than 90 mutations within *TBX5* gene include splice-site, nonsense, or frameshift mutations (6, 7). In our report, the fetus exhibited a typical HOS phenotype accompanied by a heterozygous variant, a variant inherited from the paternal variant. The paternal variant (c.510+1G>A) occurs in the splice donor, likely altering the consensus splice donor site, which caused *TBX5* gene function to be altered. The Human Splicing Finder system analysis demonstrated that the variant most probably affects splicing. To our knowledge, the variant (c.510+1G>A) is a novel variant so far, and the frequency of this mutation in the East Asian population of EXAC Normal Database was 0. According to the American College of Medical Genetics and Genomics (ACMG) standards and ACMG and ClinGen guidelines (16, 17), the variant was classified as pathogenic. Furthermore, in our case, the clinical features of the fetus completely fulfilled the strict diagnostic criteria for HOS.

In conclusion, we report a rare case of prenatal diagnosis of HOS caused by a novel mutation in the *TBX5* at 23 weeks of gestation, which will provide the basis for prenatal ultrasound diagnosis and improve the diagnostic rate.

## Data Availability Statement

The original contributions presented in the study are included in the article/supplementary material, further inquiries can be directed to the corresponding author.

## Ethics Statement

Written informed consent was obtained from the individual(s) for the publication of any potentially identifiable images or data included in this article.

## Author Contributions

GH and XW analyzed the information about patients. MK, XC, and XC collected clinical data. JZ analyzed data. NX helped to draft the manuscript and search literatures. XW designed the study. All authors read and approved the final manuscript.

## Conflict of Interest

The authors declare that the research was conducted in the absence of any commercial or financial relationships that could be construed as a potential conflict of interest.

## Publisher's Note

All claims expressed in this article are solely those of the authors and do not necessarily represent those of their affiliated organizations, or those of the publisher, the editors and the reviewers. Any product that may be evaluated in this article, or claim that may be made by its manufacturer, is not guaranteed or endorsed by the publisher.

## References

[1] Heinritz W Moschik A Kujat A Spranger S Heilbronner H Demuth S. Identification of new mutations in the TBX5 gene in patients with Holt-Oram syndrome. Heart. (2005) 91:383–4. 10.1136/hrt.2004.036855 15710732 PMC1768780

[2] McDermott DA Fong JC Basson CT. Holt-Oram Syndrome. In: GeneReviews^®^. University of Washington, Seattle, Seattle, WA (2019). p. 1993.

[3] Atik T Dervisoglu H Onay H Ozkinay F Cogulu O. A new mutation in the TBX5 gene in Holt-Oram syndrome: two cases in the same family and prenatal diagnosis. J Tropical Pediatrics. (2014) 60:257–9. 10.1093/tropej/fmt109 24408148

[4] Varela D Conceição N Cancela ML. Transcriptional regulation of human T-box 5 gene (TBX5) by bone- and cardiac-related transcription factors. Gene. (2021) 768:145322. 10.1016/j.gene.2020.145322 33221539

[5] Huang T. Current advances in Holt-Oram syndrome. Curr Opin Pediatrics. (2002) 14:691–5. 10.1097/00008480-200212000-00008 12436037

[6] Basson CT Bachinsky DR Lin RC Levi T Elkins JA Soults J. Mutations in human TBX5 [corrected] cause limb and cardiac malformation in Holt-Oram syndrome. Nat Genet. (1997) 15:30–5. 10.1038/ng0197-30 8988165

[7] Kimura M Kikuchi A Ichinoi N Kure S. Novel TBX5 duplication in a Japanese family with Holt-Oram syndrome. Pediatric Cardiol. (2015) 36:244–7. 10.1007/s00246-014-1028-x 25274398

[8] Dreßen M Lahm H Lahm A Wolf K Doppler S Deutsch MA. A novel de novo TBX5 mutation in a patient with Holt-Oram syndrome leading to a dramatically reduced biological function. Mo Genet Genomic Med. (2016) 4:557–67. 10.1002/mgg3.234 27652283 PMC5023941

[9] Vanlerberghe C Jourdain AS Ghoumid J. Holt-Oram syndrome: clinical and molecular description of 78 patients with TBX5 variants. Euro J Human Genet. (2019) 27:360–8. 10.1038/s41431-018-0303-3 30552424 PMC6460573

[10] Holt M Oram S. Familial heart disease with skeletal malformations. Br Heart J. (1960) 22:236–42. 10.1136/hrt.22.2.236 14402857 PMC1017650

[11] Nourzad G Baghershiroodi M. A case report on holt-oram syndrome (heart-hand). ARYA Atherosclerosis. (2011) 7:87–92. 22577452 PMC3347849

[12] Krauser AF Schury MP. Holt Oram Syndrome. Bangalore: StatPearls (2020). 30020711

[13] Bejiqi R Retkoceri R Maloku A Mustafa A Bejiqi R. Holt–Oram syndrome associated with complex congenital heart disease: a rare case presentation and literature review. Open Access Macedonian J Med Sci. (2020) 8:36–40. 10.3889/oamjms.2020.4287

[14] Law KM Tse KT. Prenatal sonographic diagnosis of familial Holt-Oram syndrome associated with type B interrupted aortic arch. Hong Kong Med J. (2008) 14:317–20. 18685167

[15] Varela D Varela T Conceição N. Functional analysis of two novel TBX5 variants present in individuals with Holt-Oram syndrome with different clinical manifestations. Mol Genet Genomics. (2021) 296:809–21. 10.1007/s00438-021-01781-2 33866394

[16] Zhang J Yao Y He H Shen J. Clinical interpretation of sequence variants. Curr Protocols Human Genet. (2020) 106:e98. 10.1002/cphg.98 32176464 PMC7431429

[17] Richards S Aziz N Bale S Bick D Das S Gastier-Foster J. Standards and guidelines for the interpretation of sequence variants: a joint consensus recommendation of the American College of Medical Genetics and Genomics and the Association for Molecular Pathology. Genet Med. (2015) 17:405–24. 10.1038/gim.2015.30 25741868 PMC4544753

